# Directed block copolymer self-assembly implemented via surface-embedded electrets

**DOI:** 10.1038/ncomms10752

**Published:** 2016-02-15

**Authors:** Mei-Ling Wu, Dong Wang, Li-Jun Wan

**Affiliations:** 1 Key Laboratory of Molecular Nanostructure and Nanotechnology, Institute of Chemistry, Chinese Academy of Sciences (CAS), Beijing 100190, China; 2 Beijing National Laboratory for Molecular Sciences, Beijing 100190, China; 3 University of CAS, Beijing 100049, China

## Abstract

Block copolymer (BCP) nanolithography is widely recognized as a promising complementary approach to circumvent the feature size limits of conventional photolithography. The directed self-assembly of BCP thin film to form ordered nanostructures with controlled orientation and localized pattern has been the key challenge for practical nanolithography applications. Here we show that BCP nanopatterns can be directed on localized surface electrets defined by electron-beam irradiation to realize diverse features in a simple, effective and non-destructive manner. Charged electrets can generate a built-in electric field in BCP thin film and induce the formation of perpendicularly oriented microdomain of BCP film. The electret-directed orientation control of BCP film can be either integrated with mask-based patterning technique or realized by electron-beam direct-writing method to fabricate microscale arbitrary lateral patterns down to single BCP cylinder nanopattern. The electret-directed BCP self-assembly could provide an alternative means for BCP-based nanolithography, with high resolution.

## Abstract (web summary)

The controlled orientation of block copolymers promises to deliver high resolution patterning for implementation in future nanoscale technologies. Here, the authors use the electric field from charged electrets to induce extremely localised perpendicularly oriented microdomains in polymer thin films.

## Graphical abstract

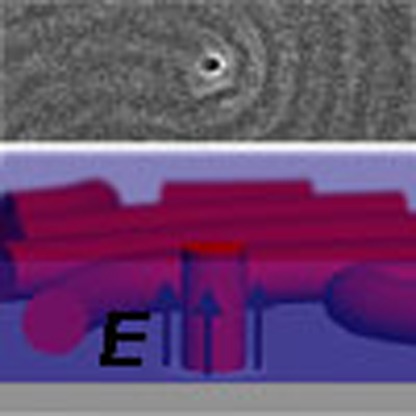

Block copolymer (BCP) self-assembly can spontaneously generate periodic arrays of microdomains with versatile morphology and nanoscale feature size in the range of ca. 10–100 nm (ref. 1). The well-defined nanostructures of BCP have been utilized as templates to control spatial order of other functional materials for a variety of applications^2^,^3^ such as microelectronic devices^4^,^5^, photovoltaic devices^6^, magnetic storage devices^7^, microreactors^8^ and porous filtration membranes^9^. In particular, BCP-based nanolithography^10^,^11^, which utilizes the self-assembled BCP thin film to pattern the substrates, has been widely recognized as a viable alternative or complementary approach to conventional photolithography^12^,^13^. It is promised to address the conflict between the continuous demanding to shrink the feature size of electronic devices and the technology and cost limits of photolithography. Owing to the favourable advantages such as high throughput, low cost and most notably, compatibility with current nanolithography streamline, BCP-based nanolithography has been targeted as one of the most important next-generation lithography techniques in the International Technology Roadmap for Semiconductors^12^.

For a typical BCP-based nanolithography, orientation of domains normal to the substrates is essential to facilitate robust pattern transfer^13^,^14^. The most common way to control the orientation of BCP microdomains is to fabricate a neutral surface through surface energy modification to balance its interfacial energy with both BCP components^15^,^16^. Taking advantage of the dielectric constant difference of each block, orientation control of the BCP self-assembly can also be achieved by applying an external electric field, although it is generally limited to BCP films with micrometre-scale thickness^17^,^18^. In addition, surface topology modulation^19^,^20^, solvent annealing^21^,^22^ and other methods^23^,^24^ have also been proposed to control BCP microdomain orientation. Besides the perpendicular orientation, controlled BCP self-assembly with lateral order and localized nanostructures^25^,^26^,^27^,^28^ is also necessary in practical lithography processes to realize pattern registration and addressable device-oriented patterns with low defects^29^,^30^. Directed self-assembly, which integrates the BCP self-assembly with the traditional lithography processes, has been developed to achieve oriented and lateral ordering of BCP films^31^,^32^ utilizing chemical epitaxy and graphoepitaxy methods^33^,^34^,^35^,^36^. In addition, electrohydrodynamic jet printing was utilized to fabricate complex and hierarchical patterns of BCP films^37^,^38^. While these methods have improved the ordering of BCP films and advanced the process of registration and addressing, they are generally subject to complex lithographic steps, limited substrates and/or physical destruction of the substrates. It would be rewarding to develop a simple, effective and non-destructive method to realize device-oriented features with versatile patterns and high nanopatterning resolution.

Herein, we introduce a novel method to achieve simultaneously orientation control and localization of BCP self-assembly implemented by surface-embedded electrets, which is fabricated by electron-beam (e-beam) irradiation. Electrets are materials that retain trapped charges or polarization, forming a quasi-permanent, macroscopic electric field around the perimeter^39^,^40^,^41^. Similar to external electric filed, which can align the BCP blocks, the electric field around the electrets is also found to be able to control the orientation of BCP film. The surface electrets-mediated BCP self-assembly provides a facile approach to BCP nanopatterning, and can yield customized nanopatterns with arbitrary geometry and high fidelity by employing an e-beam direct-writing technique. More attractively, by tuning the parameters of e-beam irradiation carefully, we can realize the formation of individual cylinder nanopattern with extremely high accuracy, which represents the utmost resolution of BCP-based nanolithography and is critical for the formation of contact and via holes in integrated circuit.

## Results

### Fabrication of nanopatterns

**Figure 1:**
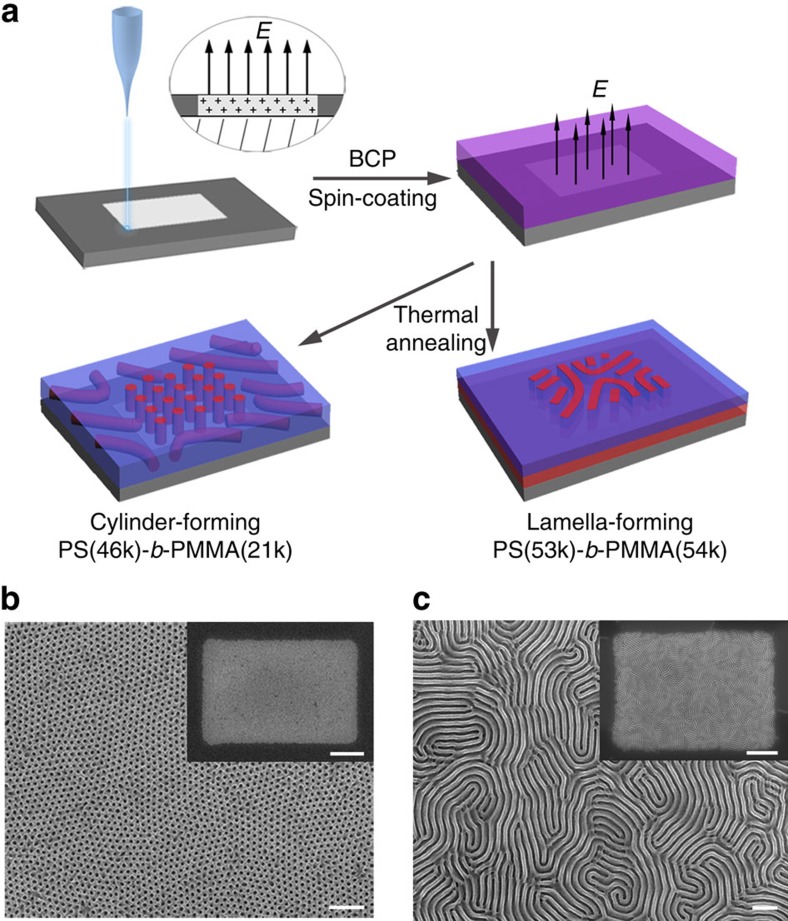
PS*-b-*PMMA self-assembly on e-beam-irradiated SiO_2_/Si substrate. (**a**) Schematic description for fabricating PS*-b-*PMMA films with perpendicular orientation on e-beam-irradiated SiO_2_/Si substrates. SEM images of PS*-b-*PMMA films self-assembled on the SiO_2_/Si wafers whose central regions were irradiated by e-beam: (**b**) Cylinder-forming PS*-b-*PMMA (46k*-b-*21k) with thickness of 37 nm and (**c**) lamella-forming PS*-b-*PMMA (53k*-b-*54k) with thickness of 56 nm (scale bars, 200 nm). The insets in **b** and **c** are the corresponding low-magnification images (scale bars, 1 μm), in which the bright contrast indicates a uniform formation of the perpendicular orientation. The samples were treated by Ar plasma to selectively remove PMMA to improve the SEM image contrast.

The overall fabricating process of electrets-induced polystyrene-b-poly(methyl methacrylate) (PS*-b-*PMMA) self-assembly is shown in Fig. 1a. Briefly, a SiO_2_/Si wafer was first irradiated by e-beam, with proper dose and then spin-coated with BCP film. After thermal annealing, the BCP film underwent microphase separation and self-assembled into stable cylindrical or lamellar nanostructures, depending on the molecular weight ratio of the blocks. Figure 1b shows the plain view scanning electron microscopy (SEM) image of a cylinder-forming BCP thin film on the SiO_2_/Si wafer, of which the central region has been irradiated by the e-beam. The as-obtained hexagonal arrays (Fig. 1b and Supplementary Fig. 1) in the irradiated region prove that microdomains of PS*-b-*PMMAs orient normal to the substrate surface. Similar results are found in the lamellar BCP film. Fingerprint arrays are formed in the irradiated area, with the lamella microdomains oriented normal to the substrate (Fig. 1c and Supplementary Fig. 2). In contrast, in the area where SiO_2_/Si wafers have not been treated by e-beam irradiation, parallel orientations are observed (Supplementary Fig. 3). To probe the internal perpendicular orientation of the entire thin films, we performed grazing-incidence small-angle X-ray scattering (GISAXS) experiment. In the two-dimensional GISAXS pattern of cylinder PS-*b*-PMMA film self-assembled on the irradiated SiO_2_/Si of ∼1 cm^2^ (Supplementary Fig. 4a), the symmetric scattering peaks are confined in the *Q*_*y*_ direction and the first-order scattering peaks at *Q*_*y*_=0.196 nm^−1^ reflect the perpendicularly oriented microdomains of the entire film with a period (domain space) *L*_0_=32.1 nm (2π/*Q*_*y*_). Similarly, perpendicular orientation of lamellae is supported by the GISAXS pattern in Supplementary Fig. 4b (*Q*_*y*_=0.119 nm^−1^ and *L*_0_=52.8 nm). In addition, the e-beam-induced BCP self-assembly is found widely feasible; all the tested PS*-b-*PMMAs of different molecular weights and block ratios show vertically oriented patterns (Supplementary Fig. 5). The above results indicate that with the aid of e-beam irradiation, we can readily achieve perpendicularly oriented microdomain of PS*-b-*PMMA films.

**Figure 2:**
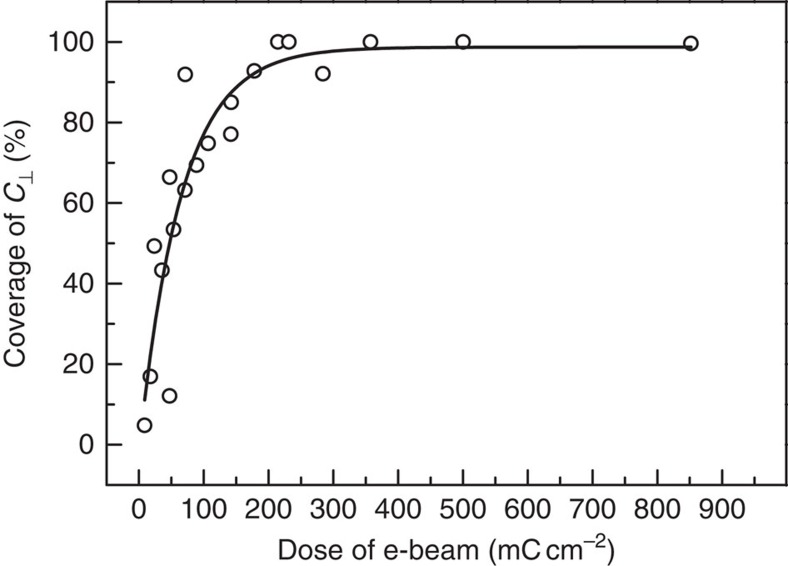
Evolution of coverage of *C*_⊥_ with e-beam dose. E-beam dose is controlled by changing irradiation area and time. Coverage of *C*_⊥_ is calculated by the area ratio of *C*_⊥_ in the whole irradiated region. The line is a fitted curve based on the scattered points. All experiments were conducted using a 16.9-pA beam current, a 5-kV accelerating voltage and PS(46k)-*b*-PMMA(21k) films with thickness of 37 nm.

The e-beam irradiation is considered critical to induce BCP orientation. To ascertain this point, we studied the influence of e-beam parameters on the self-assembly of BCP film. Here we use the area ratio of perpendicularly oriented cylinder microdomains (*C*_⊥_) in the whole irradiated region, that is, coverage of *C*_⊥_, to represent the degree of orientation. As shown in Fig. 2, the coverage of *C*_⊥_ increases gradually with e-beam dose (dose=beam current × irradiation time/area) and then stabilizes to ∼100% at an optimized e-beam dose (∼200 mC cm^−2^). A threshold e-beam dose is required to achieve 100% coverage of *C*_⊥_, which is affected by the e-beam parameters, such as beam current and accelerating voltage (Supplementary Fig. 6). The dependence of the BCP orientation on the e-beam parameters indicates that the BCP self-assembly by this method hinges largely on the surface charges induced by e-beam irradiation.

### Mechanism study

**Figure 3:**
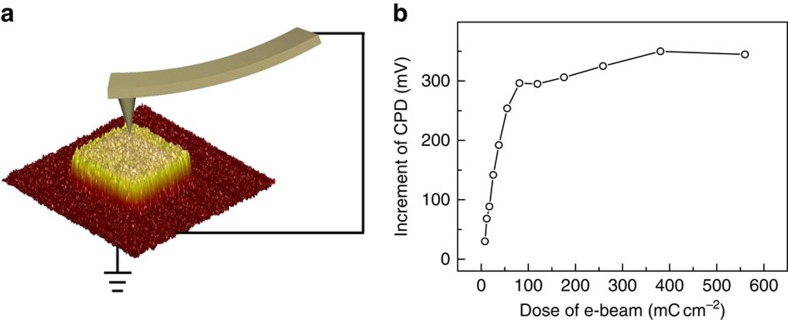
Surface potential analysis of electrets. (**a**) Schematic diagram of KPFM measurement and a representative surface potential distribution of a SiO_2_/Si surface with central region irradiated by e-beam. (**b**) Evolution of corresponding incremental CPD (ΔCPD) with e-beam dose.

The orientation dependence of PS-*b*-PMMA self-assembly on irradiation dosage provides an important cue to understand the mechanism. In this work, native layer of SiO_2_ exists on Si wafer after standard cleaning procedure (thickness: 2.3 nm). It has been well documented that SiO_2_ is an electret material that can trap charges^41^,^42^. We preformed Kelvin probe force microscope (KPFM) to trace the charges of the SiO_2_ surface, as it can measure relative surface potential and is an effective technique to uncover the charge trapping of substrates^43^. Figure 3a shows a schematic diagram of KPFM measurement and a typical KPFM mapping image of a SiO_2_/Si wafer, of which the central region has been irradiated by e-beam. According to the KPFM image, the contact potential difference (CPD, between the substrate and the tip) in the irradiated region increases significantly compared with the pristine region. The positive CPD reflects that positive charges are trapped in the SiO_2_ layer after e-beam irradiation, forming a surface-embedded electret. The CPD increment (ΔCPD) increases gradually with the e-beam dose and finally stabilizes to 300–350 mV (Fig. 3b). The evolution tendency of ΔCPD is similar to that of nanopattern coverage (Fig. 2), indicating that orientation of BCP film is closely correlated to the charge state of the surface.

In addition to KPFM measurement, SEM and X-ray photoelectronic spectroscopy (XPS) can also reflect the charging behaviour of the SiO_2_ layer. The SEM image of electret irradiated by e-beam shows higher contrast difference compared with the non-irradiated region (Supplementary Fig. 7a). The voltage contrast image is attributed to the charging of the electret^44^. XPS was performed to examine the surface constitution and chemical states of the SiO_2_/Si wafers before and after irradiation (Supplementary Fig. 7b). Binding energies of Si–Si and Si–O bonds increase gradually with the e-beam irradiation, and 0.3 eV increment of the Si–Si binding energy is found in the SiO_2_/Si substrate after irradiation (3.9 mC cm^−2^). The small positive shift of binding energy indicates the accumulation of positive charges (holes). The positive charges result from the net outcome of electron injection and secondary electron emission during irradiation. As the secondary electron emitting from the wafer occurs mainly on the surface and cannot be counteracted by the electron injection, accumulated holes are formed on the SiO_2_ layer, thereby charging the SiO_2_ electret^42^.

Earlier reports have revealed that external electric field can align the blocks of PS*-b-*PMMA along the field and control its orientation due to the dielectric permittivity difference of blocks^18^,^45^. The e-beam irradiation introduces positive charges into the surface-embedded electret and generates a static electric field *E* at the SiO_2_/Si surface (inset in Fig. 1a). For a planar electret, whose lateral dimension is much larger than its thickness *D* and the film thickness *L*, one has


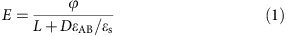


where *ϕ* is the surface potential at the electret (determined by the charges trapped in the electret), *ɛ*_AB_ is the mean (arithmetic) dielectric constant of diblock copolymer A–B (Supplementary Discussion) and *ɛ*_s_ is the dielectric constant of the electret substrate^41^. Therefore, the electric field strength is 1.5–9.5 V μm^−1^ through BCP films with thicknesses of 30–200 nm (Supplementary Discussion). These values are comparable with typical external fields (1.0–40 V μm^−1^, Supplementary Table 1) used previously in the electric field alignment of the BCP films. As the electric field is strong enough to compensate the energetic penalty from the orientation change^46^,^47^,^48^, it would induce PS-*b*-PMMA films to orient normal to the electret substrate.

It is noted that the surface energy of substrate could also be changed after e-beam irradiation. To clarify whether electric field or the surface-wetting property underlies the orientation control, a series of control experiments was conducted. A thin self-assembled monolayer of phenyltrichlorosilane was grafted onto the e-beam-irradiated SiO_2_/Si wafer, which would alter the surface energy but should not shield the electric field. As a result, on the irradiated region, PS-*b*-PMMA microdomains can still form perpendicular orientation similar to the uncoated samples, whereas parallel orientation is obtained in the non-irradiated area (Supplementary Fig. 8). Thus, the electric field is considered the driving force for BCP alignment; if the orientation control were caused by the surface energy change during e-beam irradiation, the self-assembled monolayer modification should have removed the surface energy difference at the irradiated/non-irradiated regions and the morphology of BCP film should be the same. Furthermore, we removed the charges in the SiO_2_/Si electrets deliberately by thermal annealing (>500 °C; electrets are relatively stable at ∼250 °C) or by piranha solution cleaning before PS*-b-*PMMA self-assembly. Parallel orientation is obtained on the SiO_2_/Si without the electric field alignment (Supplementary Fig. 9). Third, we have extended the present method to achieve perpendicular orientation of BCP film on other electret substrates. Uniform vertical cylindrical PS-*b*-PMMA films are attained on various e-beam-irradiated electrets, including SiO_2_/Si, PI and Si_3_N_4_/SiO_2_/Si (nitride–oxide–silicon (NOS); Supplementary Fig. 10). Intriguingly, wettability experiment shows that charged SiO_2_/Si is preferential to PMMA block, charged PI is relatively preferential to PS block and charged NOS is non-preferential for both blocks (see Supplementary Table 2 and Supplementary Discussion for more discussion). Perpendicular orientation of PS-*b*-PMMA film is successfully achieved regardless of the preferential wetting properties (SiO_2_/Si, PI). These results further prove that the electric field from the charged surface electret is the critical driving force for the perpendicular orientation of PS-*b*-PMMA films.

### Features and merits

As the directed self-assembly of PS*-b-*PMMA film is implemented with surface-embedded electrets, this method exhibits several pronounced advantages. First, this method is feasible, requiring virtually no chemical pretreatments, and is applicable to many electret materials. As discussed above, other electret substrates besides SiO_2_ are suitable for this method. In addition, this method is applicable to SiO_2_ layer with a wide thickness range (Supplementary Fig. 11).

In the conventional approaches to control the BCP orientation by surface energy modification, the BCP thickness is generally limited to below ∼3 *L*_0_ in PS-*b*-PMMA. For thicker films, driving force from neutral interfaces declines and the vertical microdomains would not penetrate through the entire film^10^,^49^. External electric field-based alignment method is applicable for film thickness of micrometres, but it is difficult to be applied to BCP with nanometre scale, as BCP film with sufficient thickness is required for electrode separation^31^. In contrast, the built-in electric field originated from the surface electret eliminates the limitation of external electric field to apply to film thickness down to tens of nanometres. As shown in Supplementary Fig. 12, the PS-*b*-PMMA films of different film thicknesses up to 161 nm can orient normal to surface of the electret and the degree of long-range lateral order slightly increases with the film thickness. We can further fabricate PS(53k)-*b*-PMMA(54k) film with thickness of 208 nm (∼3.9 *L*_0_), with vertical orientation penetrating the entire film thickness (Supplementary Fig. 13a). Moreover, in the GISAXS pattern (Supplementary Fig. 13b), the pronounced first-order peak at *Q*_*y*_=0.12 nm^−1^ and the high-order peaks with relative *Q*_*y*_ ratios of 1:2:3:4 also evidence that the lamella BCP microdomains orient normal to the substrate throughout the entire film.

The electrets-mediated BCP orientation can yield nanopatterns in millimetre scale (mm scale) and produce customized patterns. By increasing e-beam diameter and beam current, the process of e-beam irradiation can be significantly accelerated. For example, when e-beam of 20 μm in diameter (see Mode 3 in the Method) is employed, the irradiation speed increases from 0.4 to 2.5 × 10^3^ μm^2^ s^−1^ and it takes ∼30 min to irradiate a region of 3.0 × 2.2 mm^2^. As shown in Supplementary Fig. 14, perpendicularly oriented cylinder morphology is realized in several square millimetres with high uniformity. The orientation quality of the mm-scale BCP film is comparable to those fabricated by surface energy modification, surface topology modulation and external electric alignment^15^,^18^,^50^. In addition, the electret implantation can be guided by customized masks to realize patterned BCP orientation in mm scale (see Supplementary Fig. 15 for the process and pattern images).

**Figure 4:**
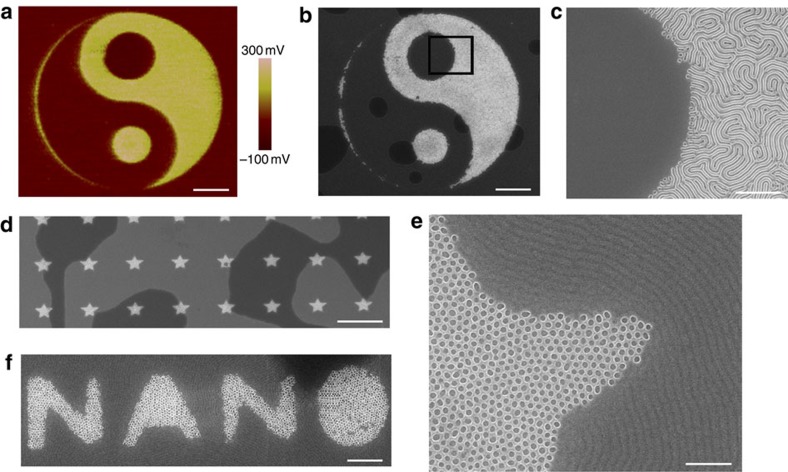
BCP nanopatterns with user-defined diagrams. (**a**) Surface potential image of a Tai Chi diagram fabricated by e-beam irradiation with EBD. (**b**,**c**) SEM images of the corresponding self-assembled morphology of PS(53k)-*b*-PMMA(54k) film (thickness: 56 nm). Spatially oriented PS(46k)-*b*-PMMA(21k) film with various geometries: (**d**) patterned array of stars; (**e**) magnified view of **d**; (**f**) pattern of letters NANO. The film thickness in **d**,**e** and **f** is 37 nm. Scale bars, 2 μm (**a**,**b**); 500 nm (**c**); 5 μm (**d**); 200 nm (**e**); 500 nm (**f**).

Finally, the electret charging can be realized by the technique of e-beam direct writing (EBD) for patterns with precise size and customized geometry. EBD technique has been extensively applied in fabricating patterned templates as a mask-free method. By exploiting EBD to manipulate the e-beam moving path, we can fabricate e-beam-irradiated region with arbitrary geometries and sizes. The fine and arbitrary features of EBD endow this method with splendid abilities to produce versatile user-defined layouts for localized and self-registered BCP nanopatterns. We elucidate this combined technique with a pattern of Tai Chi diagram. The Tai Chi-patterned electret was first fabricated by EBD, which was demonstrated by the KPFM image (Fig. 4a). After BCP self-assembly on the customized electret pattern, Tai Chi patterns can be easily built with perpendicularly oriented lamella microdomains in high fidelity (Fig. 4b,c and Supplementary Fig. 16). Using BCP with different molecular weight ratios, we can form nanopattern of fingerprints and dots in Yin and Yang deliberately (see Supplementary Fig. 16 for more nanopatterns with Tai Chi diagram). Furthermore, patterns of stars and word ‘NANO' with perpendicular orientation of cylinder-forming BCP can be also fabricated successfully by a similar method (Fig. 4d–f).

**Figure 5:**
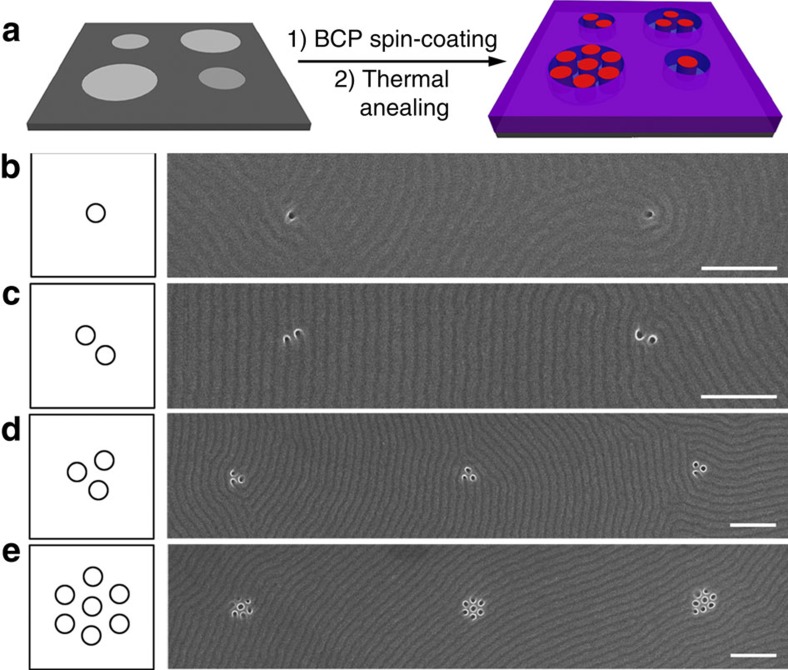
High-resolution BCP patterns realized by e-beam control. (**a**) Schematic process of high-resolution perpendicular cylindrical BCP nanopattern self-assembled on charged electrets with controlled circular size and e-beam dose. (**b**–**e**) SEM images of individual hole and patterned holes of PS-*b*-PMMA film after PMMA-block removal (scale bars, 200 nm). Each hole corresponds to the position of a vertically oriented PMMA microdomain of PS-*b*-PMMA film. The film thickness is 37 nm.

Intriguingly, we further demonstrate orientation control over individual cylinders, which represents the ultimate resolution limit for directed BCP self-assembly, as a most attractive feature by controlling the size of the surface-embedded electrets with a highly focused e-beam. Patterns of isolated hole or several holes can be formed with an irradiated circle region by careful e-beam manipulation (Fig. 5). Highly focused e-beam can precisely control the spatial distribution of the electret, at surface of which local electric field is strong enough to control the orientation of PS-*b*-PMMA for nucleation in a minimum volume^51^. As shown in Fig. 5b, individual cylinders can be realized on the SiO_2_ surface electret obtained from superfine e-beam irradiation. The control over individual microdomain of BCP self-assembly, as the resolution limit of the BCP nanopattern, is of paramount importance for application as contact holes in integrated circuit^12^. Moreover, the high-resolution nanopattern implemented on the localized electrets is free from small guiding templates or physical destruction of the substrate, and thus remarkably facilitates the process of subsequent high-resolution nanopattern transfer to other substrates^35^. Increasing the irradiated size or e-beam dosage, several cylinders are achieved in a controllable manner (Fig. 5c–e). An array composed of ordered vertical cylinder can also be successfully fabricated on an irradiated linear region (Supplementary Fig. 17a). When applying a lamella-forming polymer to such linear electret, vertical lamellae orient normal to the domain boundary (Supplementary Fig. 17b) for minimizing the free energy^52^. We can envision that the approach of surface electret-directed BCP self-assembly holds feasibility and opportunity to form accurate registering patterns provided with ultra-high-resolution e-beam^53^.

## Discussion

We have experimentally demonstrated the mechanism and appealing merits of the electret-mediated BCP self-assembly. The charged electret introduces an electric field that aligns the BCP film. In the electric field *E* over the electret, diblock copolymer A–B consisting of blocks with different dielectric constants (*ɛ*_A_ and *ɛ*_B_) is aligned by the driving force^47^,^54^


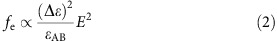


where Δ*ɛ*=|*ɛ*_A_−*ɛ*_B_| is the dielectric constant contrast of blocks A and B, and *ɛ*_AB_ is the dielectric constant of A–B. The microdomains would be aligned parallel to the electric field (that is, perpendicular to the substrate) as long as the electrostatic energy (*F*_e_), which favours the perpendicular orientation of BCP, overcomes the energy penalty from the BCP orientation transformation (*F*_p_, mainly the elastic free energy and interfacial energy). Under a critical condition, the change of free energy (Δ*F*_e_ and Δ*F*_p_) of BCP film from parallel to perpendicular orientation equals 0, that is,


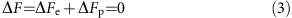


The critical surface potential (*ϕ*_c_) can be derived from equation (3), which is the threshold of surface potential to compensate the surface energy penalty and provides a theoretical assessment of possibility to align the BCP via the charged electret. Key parameters influencing *ϕ*_c_ include dielectric constants (*ɛ*_A_ and *ɛ*_B_), BCP film thickness *L* and interfacial energy mismatch *δ*. On the basis of the lamellar diblock copolymer A–B (in a strong-segregation regime) and a planar electret model, the dependence of *ϕ*_c_ on these parameters is theoretically calculated and plotted in Supplementary Fig. 18 (see Supplementary Discussion for details). According the theoretical estimation, the influence of dielectric constant contrast Δ*ɛ* (electrically relative) is significant, whereas the effect of interfacial energy is minor. The interfacial energy influences the BCP self-assembly by changing the threshold of the surface potential. The dependence of the electret-directed self-assembly on the film thickness is also insignificant.

In this study, the surface electrets were charged via e-beam irradiation to produce BCP nanopatterns with sizes ranging from nanoscale to mm scale. In fact, electrets can also be charged via corona discharge or a metal-coated polydimethylsiloxane stamp, to yield inch-scale built-in electric field, which is promising to make the electrets-aided patterning practically scalable. It is noteworthy that though this BCP self-assembly method applying local electric field can effectively achieve spatial orientation control of PS-*b*-PMMA film, further work is necessary to control the defects and achieve highly ordered BCP assembly.

In summary, we have presented a novel approach to achieve orientation control of the self-assembled PS*-b-*PMMA film with the aid of surface electret. The surface-embedded electret, which is charged by e-beam irradiation, generates static electric field in polymer film and control the orientation of BCP film perpendicular on the surface. This method is marked with simplicity and compatibility. Various customized patterns are achievable with size ranging from millimetre with a mask-based patterning technique, to nanometre or micrometre integrated with EBD. By precisely tuning the e-beam parameters, we demonstrate the formation of individual cylinder nanopattern, which represents the resolution limit of BCP nanopattern and provides promising prospect in achieving accurate registering.

## Methods

### Cleaning of substrates

Silicon wafers were cleaned in piranha solution at boiling water bath for 30 min and then rinsed with super-pure water. Polyamide film was cleaned in ethanol and acetone solution with ultrasonic cleaner. Si substrate with Si_3_N_4_/SiO_2_ (2.3 nm/2.3 nm) double layer (NOS) was fabricated by magnetron sputtering and was directly used. All other substrates were blow-dried with nitrogen flow before use.

### E-beam irradiation and post treatment

Three e-beam irradiation modes were used in this work for different experimental purposes.

Mode 1: E-beam irradiated in repeated raster scan mode, producing numerous frames. The scan time for each frame was 472 ms. Each scan consisted of 768 × 512 pixels, and the irradiation time was 1 μs for each pixel. Diameter of the e-beam was ∼5 nm. E-beam dose was determined by beam current, irradiation time and irradiation area.

Mode 2: E-beam irradiated in single-frame mode (the same as EBD technique). E-beam dose was user-defined. The point-to-point spacing was 4.6 nm. Diameter of the e-beam was ∼5 nm.

Mode 3: E-beam irradiation is the same as that in Mode 1, except that the e-beam diameter was ∼20 μm.

Irradiation process in Modes 1 and 2 were performed on Helios NanoLab 600i (FEI company, Germany). The chamber pressure was ∼2.0 × 10^−4^ Pa and the working distance was 4 mm. Irradiation process in Mode 3 was performed on the electron microprobe (Shimadzu Corp., Japan). The chamber pressure was ∼2.0 × 10^−4^ Pa and the working distance was 5 mm.

Modes 1 and 2 were used to fabricate small-size or patterned irradiation region. Beam current and accelerating voltage can be adjusted as required. Unless otherwise stated, the beam current was 16.9 pA and the accelerating voltage was 5 kV. Beam current was measured accurately by a Faraday cup. To form the isolated or superfine patterns, e-beam dose was ∼50 mC cm^−2^ and the irradiated region was set as a circle with a diameter of 50–100 nm.

Mode 3 was used to form large-area irradiation region (mm scale). The beam current was 100 nA and the accelerating voltage was 15 kV. The SiO_2_/Si wafer was irradiated for 30 min to form an irradiated region of 3 × 2.25 mm^2^. To achieve patterned irradiation, Cu grid was used as a mask to block partial e-beam irradiation.

To study the post-treatment effects on the BCP self-assembly, SiO_2_/Si substrates irradiated by Mode 1 were annealed at 250 or 500 °C for 2 h in Ar flow or immersed in piranha solution (H_2_SO_4_/H_2_O_2_ mixture) at 100 °C for 0.5 h. To alter the surface energy of SiO_2_/Si, SiO_2_/Si substrate irradiated by Mode 1 was immersed in phenylteichlorosilane/toluene mixture (1 vol%) for 0.5 h and was then washed in toluene to remove the unreacted silane.

### Formation of PS-*b*-PMMA film

Cylinder-forming PS*-b-*PMMAs (46k-*b*-21k, 68k-*b*-33.5k and 35k-*b*-12.5k g mol^−1^) and lamella-forming PS*-b-*PMMAs (53k-*b*-54k and 23k-*b*-22k g mol^−1^) diblock copolymers were purchased from Polymer Source. The BCPs were dissolved in toluene and spin-coated onto the irradiated SiO_2_/Si wafers. Unless otherwise stated, the film thickness of PS-*b*-PMMA (46k-*b*-21k) and PS-*b*-PMMA (53k-*b*-54k) was 37 and 56 nm, respectively, which was determined by a spectroscopic ellipsometer (SE 850 DUV, SENTECH Instruments). The BCP films were annealed at 250 °C for 2 h in Ar flow for microphase separation.

### KPFM measurement

The KPFM measurement was operated on Multimode 8 (Bruker Corp.). The samples were prepared with the Helios 600i system, and a marker was added near the irradiated zone to locate the irradiated zone quickly under the charge-coupled device of KPFM system. We used Si probe with 1.5 N m^−1^ force constant and 250 kHz resonance frequency. Scan velocity was 0.6 Hz and number of scan lines was 512. All KPFM images were analysed using Nanoscope Analysis software.

### Characterization

The surface morphology of the BCP films was observed by Helios 600i SEM system with an accelerating voltage of 5 kV. To increase the contrast of SEM image, all the annealed samples were treated with Ar plasma (power, 50 W; flow rate, 20 s.c.c.m.) to remove the PMMA domains selectively. GISAXS experiments were conducted at beamline 16B of the Shanghai Synchrotron Radiation Facility. The synchrotron X-ray energy was 10 keV and sample-to-detector distance was 1,820 mm. The incident angle of X-ray beam was 0.185°. A vacuum guide tube in which the scattered beam passed through was used to minimize air scattering. The two-dimensional GISAXS patterns were recorded on a Mar 165 charge-coupled device detector (2,048 × 2,048 pixels, 80 μm per pixel). The surface elemental information was analysed by the X-ray photoelectron spectroscopy on the Thermo Scientific ESCALab 250Xi using 200 W Al Kα radiation. The irradiated SiO_2_/Si wafers for GISAXS and XPS measurement were prepared by Mode 3.

## Additional information

**How to cite this article**: Wu, M.-L. *et al.* Directed block copolymer self-assembly implemented via surface-embedded electrets. *Nat. Commun.* 7:10752 doi: 10.1038/ncomms10752 (2016).

## Supplementary Material

Supplementary Information Supplementary Figures 1-18, Supplementary Tables 1-2, Supplementary Discussion and Supplementary References
