# Supplementary material for: Directed block copolymer self-assembly implemented via surface-embedded electrets
Source: Nat Commun. 2016 Feb 15;7:10752. doi: 10.1038/ncomms10752 (PMC4756386; doi:10.1038/ncomms10752)
Supplement: Supplementary Information — Supplementary Figures 1-18, Supplementary Tables 1-2, Supplementary Discussion and Supplementary References [file ncomms10752-s1.pdf]

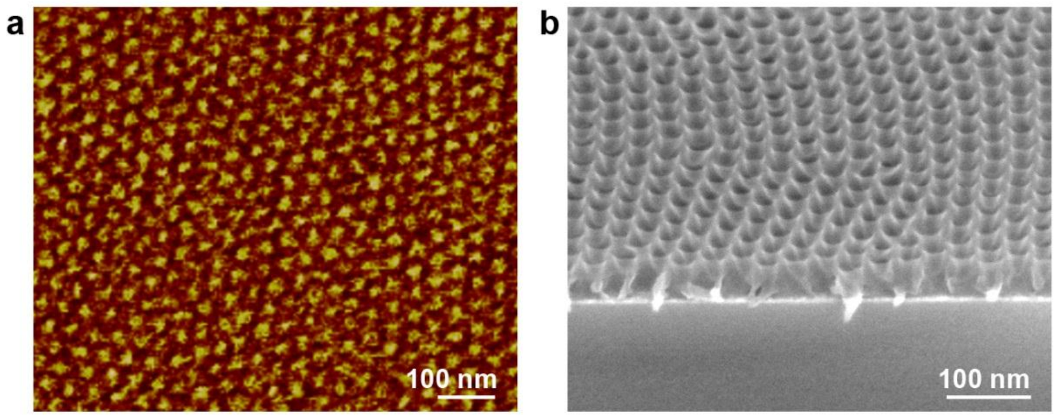

Supplementary Figure 1 | Morphology of PS(46k)-*b*-PMMA(21k) self-assembled on a SiO_2/Si substrate irradiated by e-beam. (a) AFM phase image without removing PMMA domains (film thickness: 37 nm); (b) side-view (52 °) SEM image with PMMA removed.

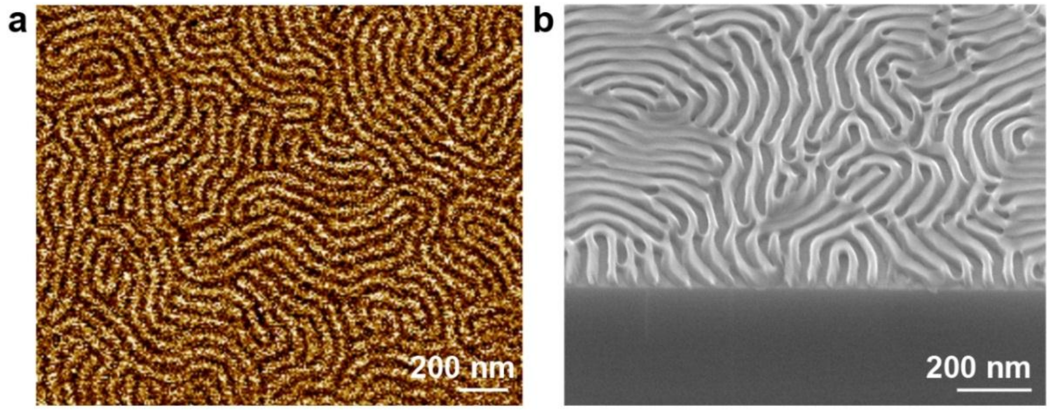

Supplementary Figure 2 | Morphology of PS(53k)-*b*-PMMA(54k) self-assembled on a SiO_2/Si substrate irradiated by e-beam. (a) AFM phase image without removing PMMA domains (film thickness: 56 nm); (b) side-view (52 °) SEM image with PMMA removed.

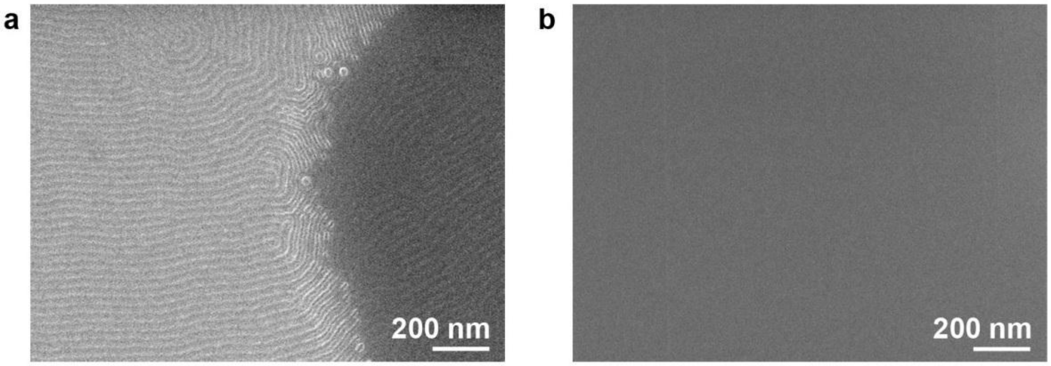

Supplementary Figure 3 | SEM images of PS-*b*-PMMAs self-assembled on SiO_2/Si substrates without e-beam irradiation. PS-*b*-PMMA films are (a) 46k-*b*-21k (film thickness: 37 nm), (b) 53k-*b*-54k (film thickness: 56 nm). The BCP films with parallel orientation are formed on the non-irradiated SiO_2/Si substrates because of the preferential wetting surface of SiO_2/Si substrates.

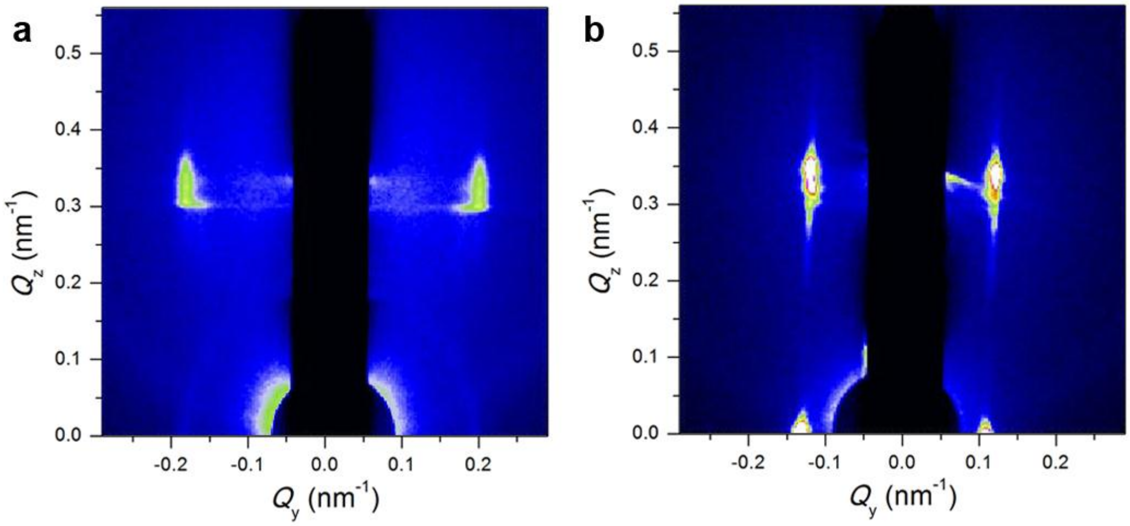

Supplementary Figure 4 | 2D GISAXS patterns of PS-*b*-PMMA self-assembled on a SiO_2/Si substrate irradiated by e-beam. PS-*b*-PMMA films are (a) 46k-*b*-21k (film thickness: 37 nm), (b) 53k-*b*-54k (film thickness: 56 nm). The BCP films were approximately 1 cm^2 and were not treated by Ar plasma.

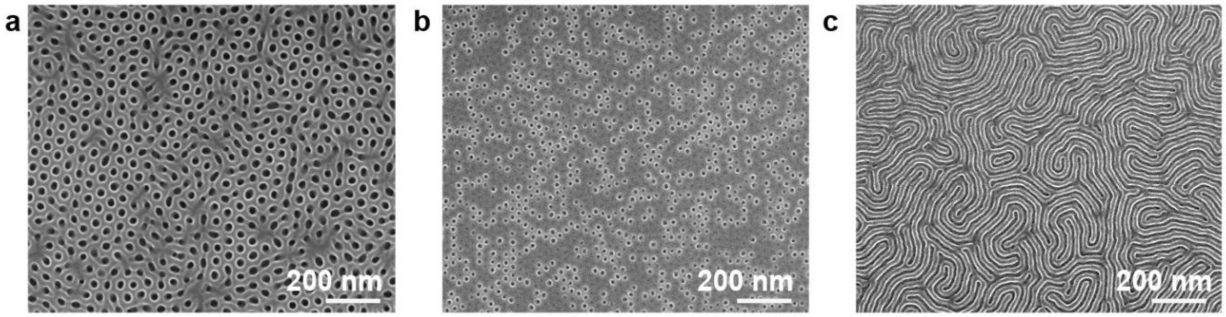

Supplementary Figure 5 | SEM images of PS-*b*-PMMAs self-assembled on SiO_2/Si substrates after e-beam irradiation. PS-*b*-PMMA films are (a) 68k-*b*-33.5k, (b) 35k-*b*-12.5k, and (c) 23k-*b*-22k. Film thicknesses are 64, 27, and 28 nm, respectively.

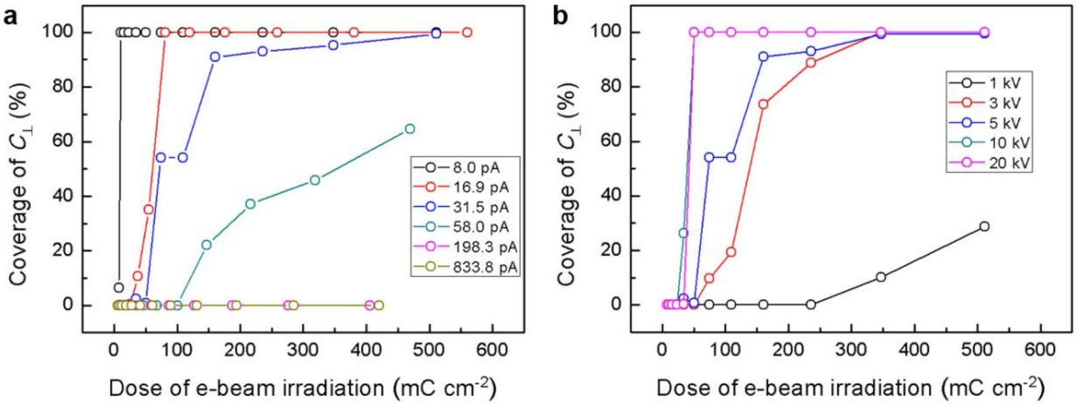

Supplementary Figure 6 | Influence of e-beam parameters on BCP self-assembly. Evolution of coverage of $C_\perp$ with e-beam dose at (a) different beam currents and (b) different accelerating voltages.

A threshold e-beam dose is required to achieve 100% coverage of $C_\perp$. As shown in the figure, the threshold of e-beam dose is found to increase with the beam current and decrease with the accelerating voltage. Because the beam current increases electron injection and counteracts the accumulated holes, it raises the threshold of e-beam dose. The accelerating voltage, on the contrary, increases the probability of secondary electron emission and produces more holes, thus reducing the threshold dose.

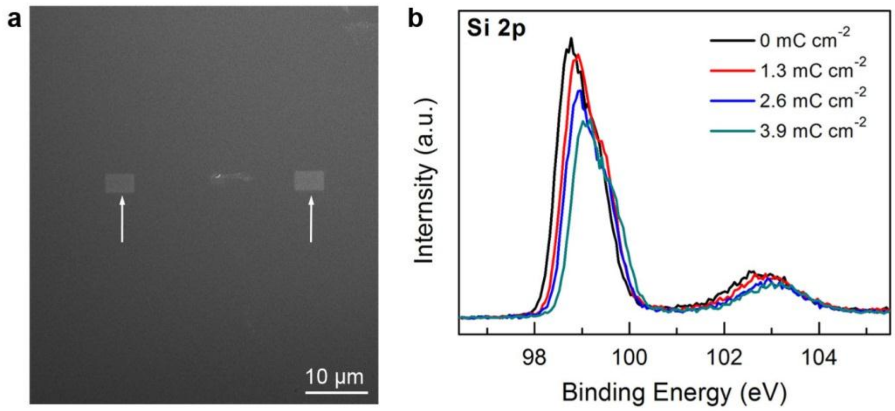

Supplementary Figure 7 | Characterization of SiO_2/Si wafer irradiated by e-beam. (a) SEM image of SiO_2/Si wafer with partial area irradiated by e-beam (indicated by the arrows); (b) Si 2p XPS spectra of SiO_2/Si wafers irradiated by e-beam with different doses. To prepare the sample for XPS, Si wafers of ~3 × 2.25 mm^2 were irradiated by electron probe as described in Mode **3**.

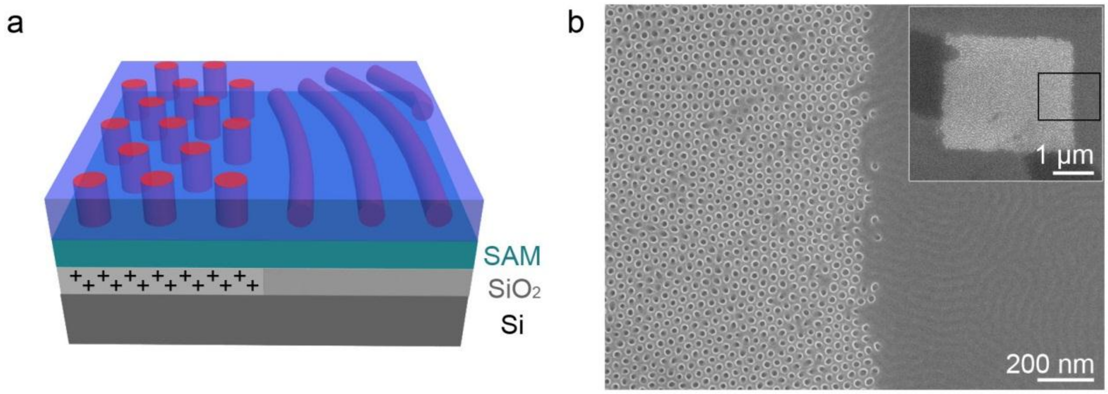

Supplementary Figure 8 | BCP self-assembled on a phenyltrichlorosilane modified SiO_2/Si electret. (a) Schematic image of the treatment to the SiO_2/Si substrate. The SiO_2/Si substrate was locally treated by e-beam irradiation before modified by phenyltrichlorosilane over the whole wafer. (b) SEM image of PS(46k)-*b*-PMMA(21k) morphology at the boundary between the irradiated (left) and non-irradiated (right) region (inset: low-magnification image).

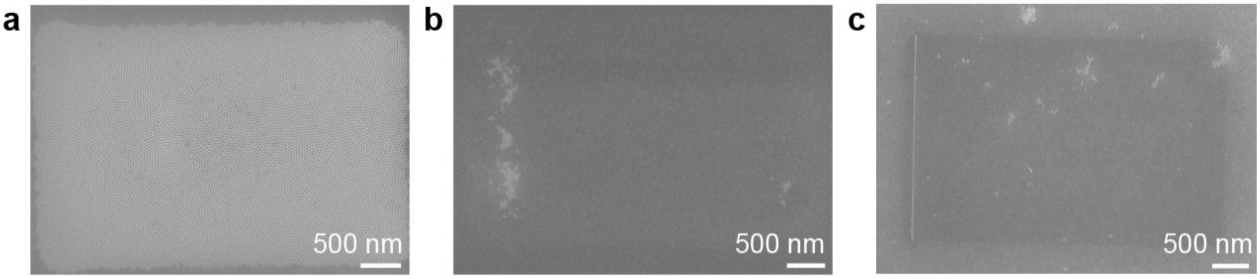

Supplementary Figure 9 | Morphology of PS-*b*-PMMA self-assembled on charge-removed SiO_2/Si substrate. SEM images of PS(46k)-*b*-PMMA(21k) (film thickness: 37 nm) self-assembled on charged SiO_2/Si electrets that were post-treated by (a) thermal annealing at 250 ℃ for 2 h in Ar flow, (b) thermal annealing at 500 ℃ for 2 h in Ar flow, (c) piranha solution cleaning. Excessive thermal annealing in 500 ℃ weakens the electric field significantly and thus perpendicular orientation could not be formed in the irradiated region.

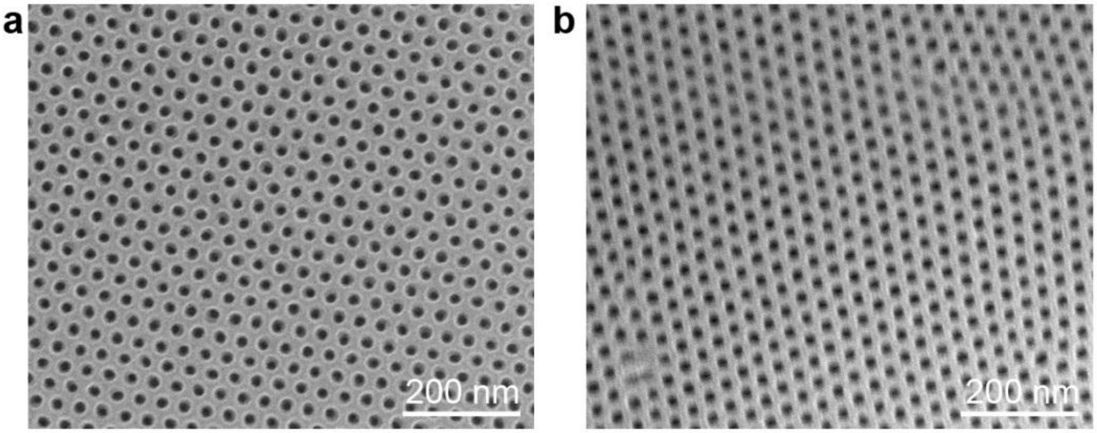

Supplementary Figure 10 | PS-*b*-PMMA self-assembled on other electrets. SEM image of PS(46k)-*b*-PMMA(21k) (film thickness: 37 nm) self-assembled on electrets irradiated by e-beam: (a) Si_3N_4/SiO_2/Si substrate, (b) polyimide film.

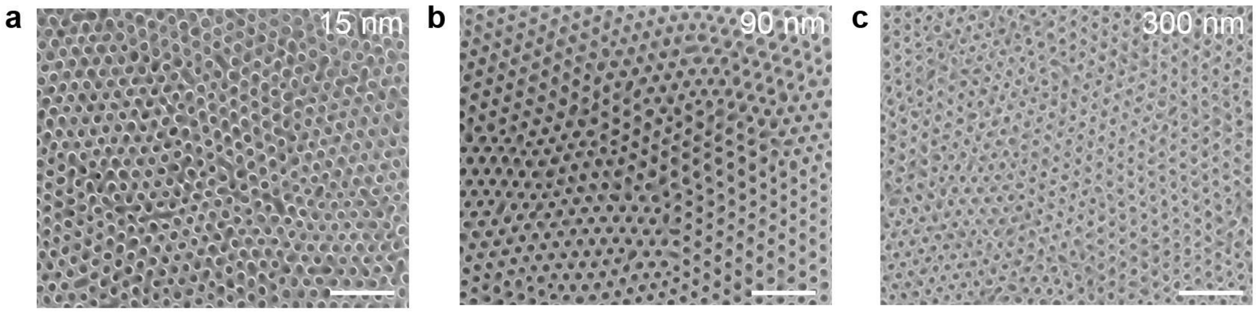

Supplementary Figure 11 | Influence of SiO_2 layer thickness on the self-assembly morphology of PS-*b*-PMMA on electrets. SEM images of PS(46k)-*b*-PMMA(21k) (film thickness: 37 nm) self-assembled on SiO_2/Si electret with different SiO_2 layer thicknesses (e-beam dose: 50 mC cm^{-2}; scale bars: 200 nm). SiO_2 layers with different thicknesses were obtained by thermal oxidation of Si wafers at 900 ℃ for 10–120 min.

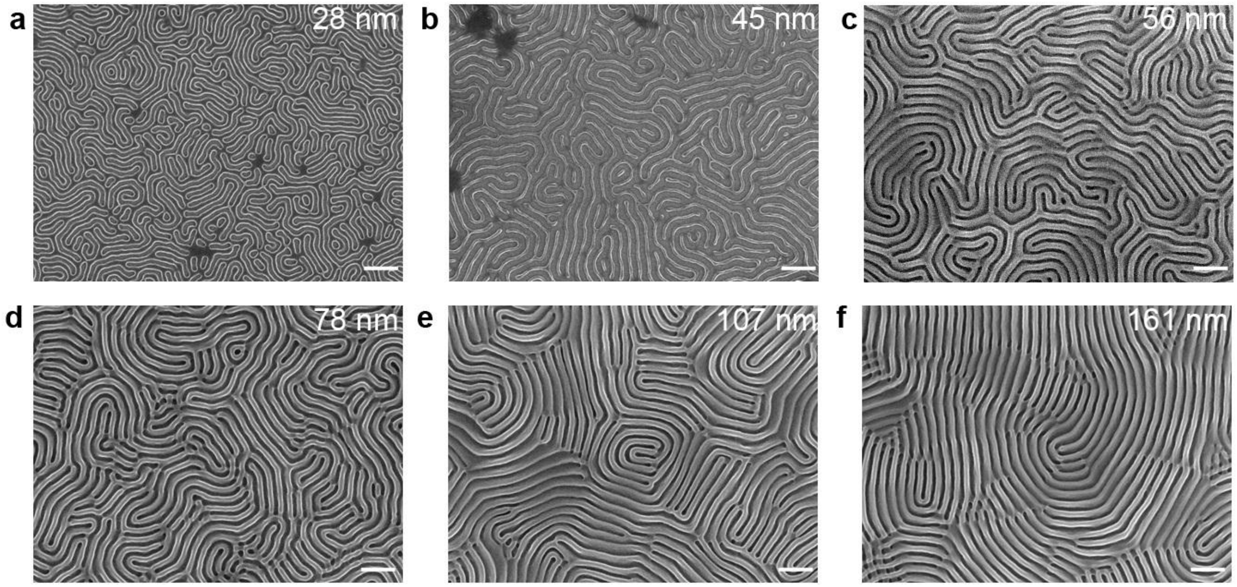

Supplementary Figure 12 | Influence of BCP film thickness on the self-assembly morphology of PS-*b*-PMMA on electrets. SEM images of PS(53k)-*b*-PMMA(54k) with different film thicknesses self-assembled on a SiO_2/Si electret (SiO_2 thickness: 2.3 nm) with an e-beam dose of 50 mC cm^{-2} (scale bars: 200 nm). BCP films with different thicknesses were obtained from different BCP concentrations (6–30 mg mL^{-1}).

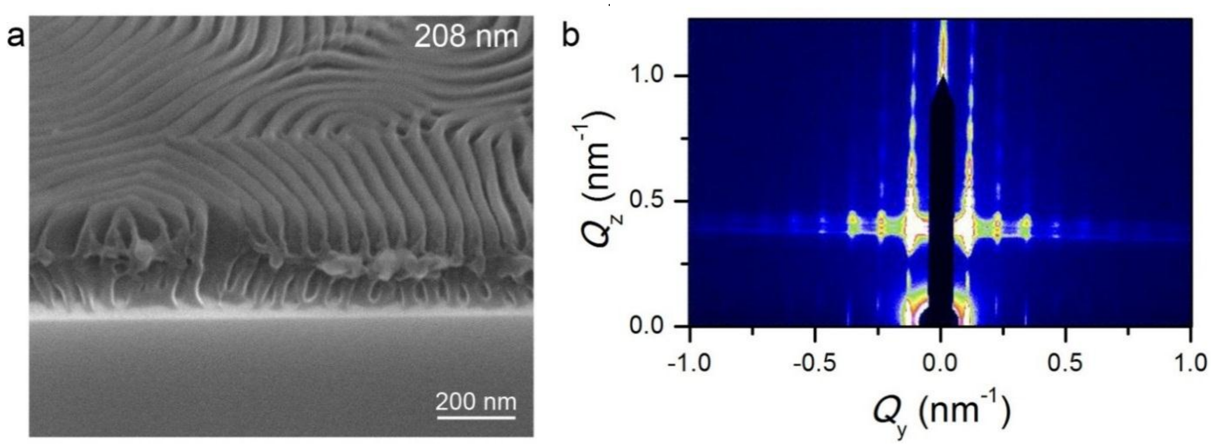

Supplementary Figure 13 | Perpendicularly orientated microdomain of PS-*b*-PMMA film with thickness of 208 nm on a SiO2/Si electret. (a) Side-view (52°) SEM image and (b) two-dimensional GISAXS patterns of PS(53k)-*b*-PMMA(54k) film on a SiO_2/Si electret (SiO_2 thickness: 2.3 nm). The samples have been treated with Ar plasma. The area of the film for GISAXS experiment was approximately 1 cm^2.

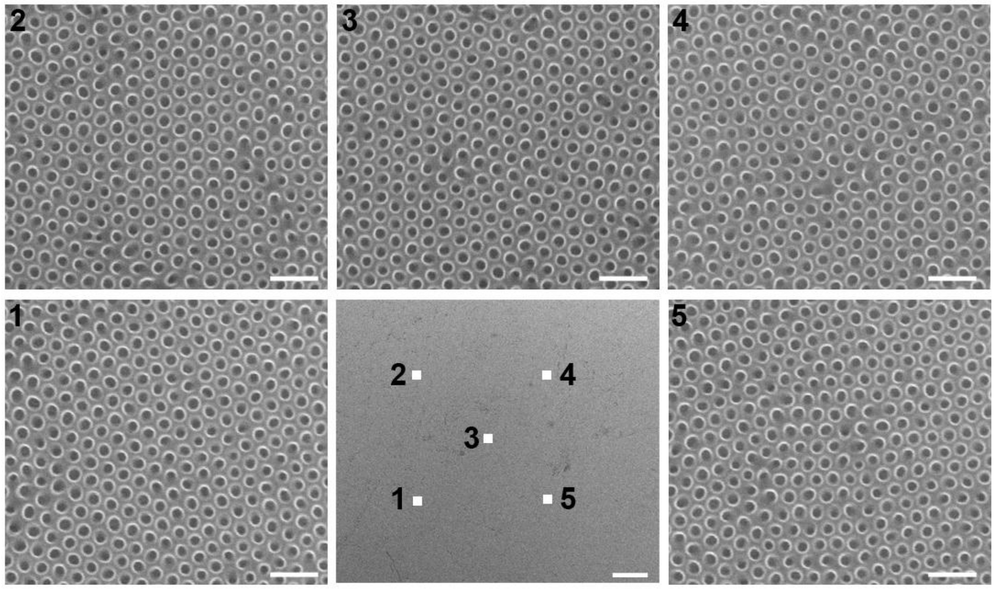

Supplementary Figure 14 | Millimetre-scale self-assembled BCP film. SEM image of a self-assembled PS(46k)-*b*-PMMA(21k) film in mm-scale (scale bar: 200 μm) and high-magnification images at Points 1–5 (scale bars: 100 nm).

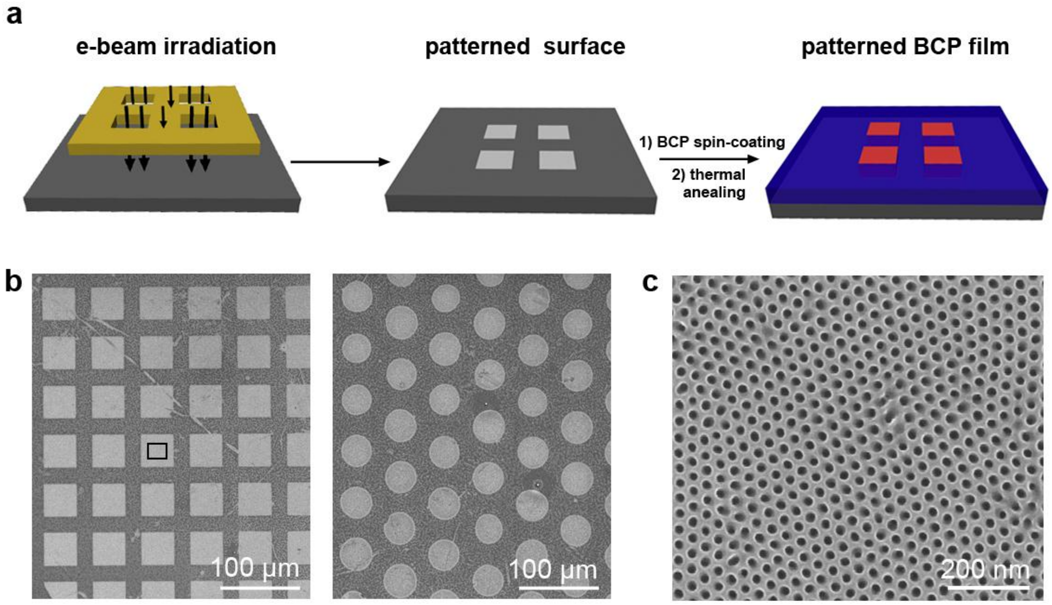

Supplementary Figure 15 | Patterned BCP films fabricated from Cu masks based patterning technique. (a) Schematic process of forming patterned BCP film with a mask, (b) self-assembled PS(46k)-*b*-PMMA(21k) films with patterns obtained with Cu grid masks composed of square or circular holes, (c) high-magnification image of rectangle region in (b). Film thickness: 37 nm.

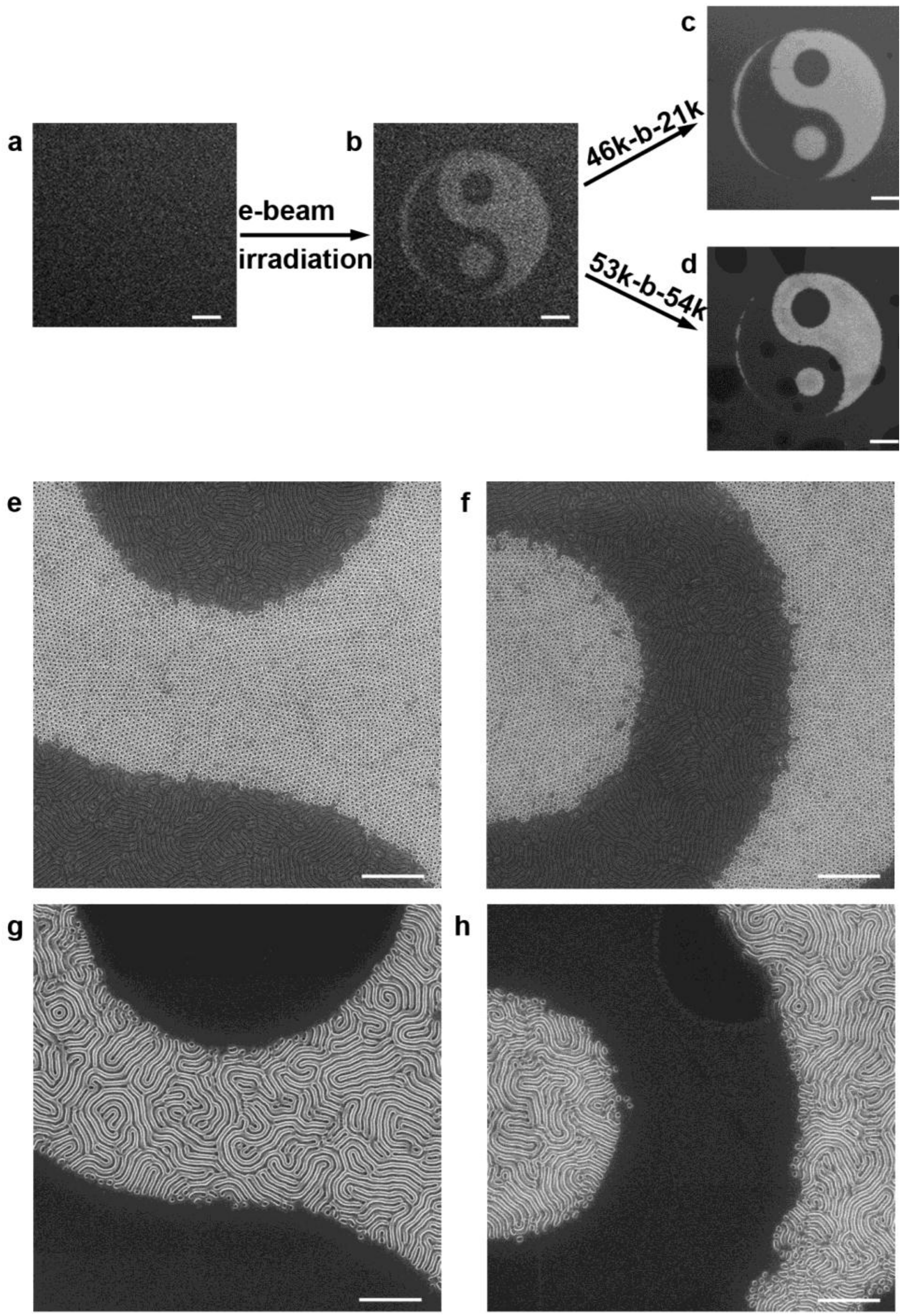

Supplementary Figure 16 | BCP self-assembled nanopattern with a Tai Chi diagram. SEM images of (a) pristine SiO_2/Si wafer, (b) charged SiO_2/Si electret with a Tai Chi diagram, (c, e, f) self-assembled morphology of PS(46k)-*b*-PMMA(21k) (film thickness: 37 nm) and (d, g, h) PS(53k)-*b*-PMMA(54k) (film thickness: 56 nm) on the SiO_2/Si electret. Scale bars: 1 μm (a–d), 500 nm (e–h).

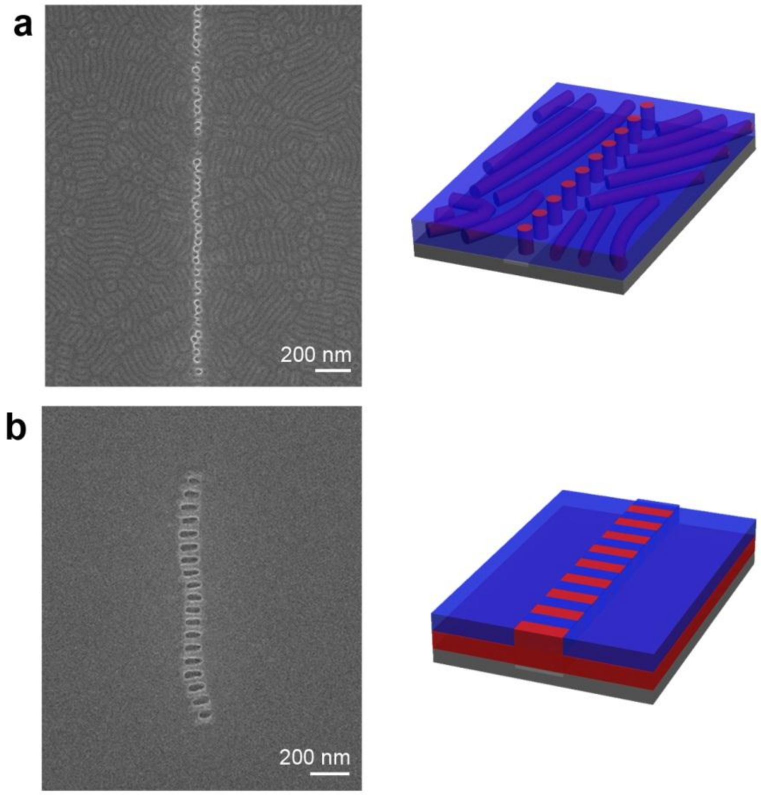

Supplementary Figure 17 | Self-assembled BCP superfine morphology on an irradiated linear region. SEM images and schematic diagrams of (a) array consisting of individual vertical cylinders of PS(46k)-*b*-PMMA(21k) (film thickness: 37 nm; each hole contains one microdomain of PS-*b*-PMMA), (b) stripe formed by vertical lamellae of PS(53k)-*b*-PMMA(54k) (film thickness: 56 nm; each vertical lamella linear orients normal to the long axis of domain boundary).

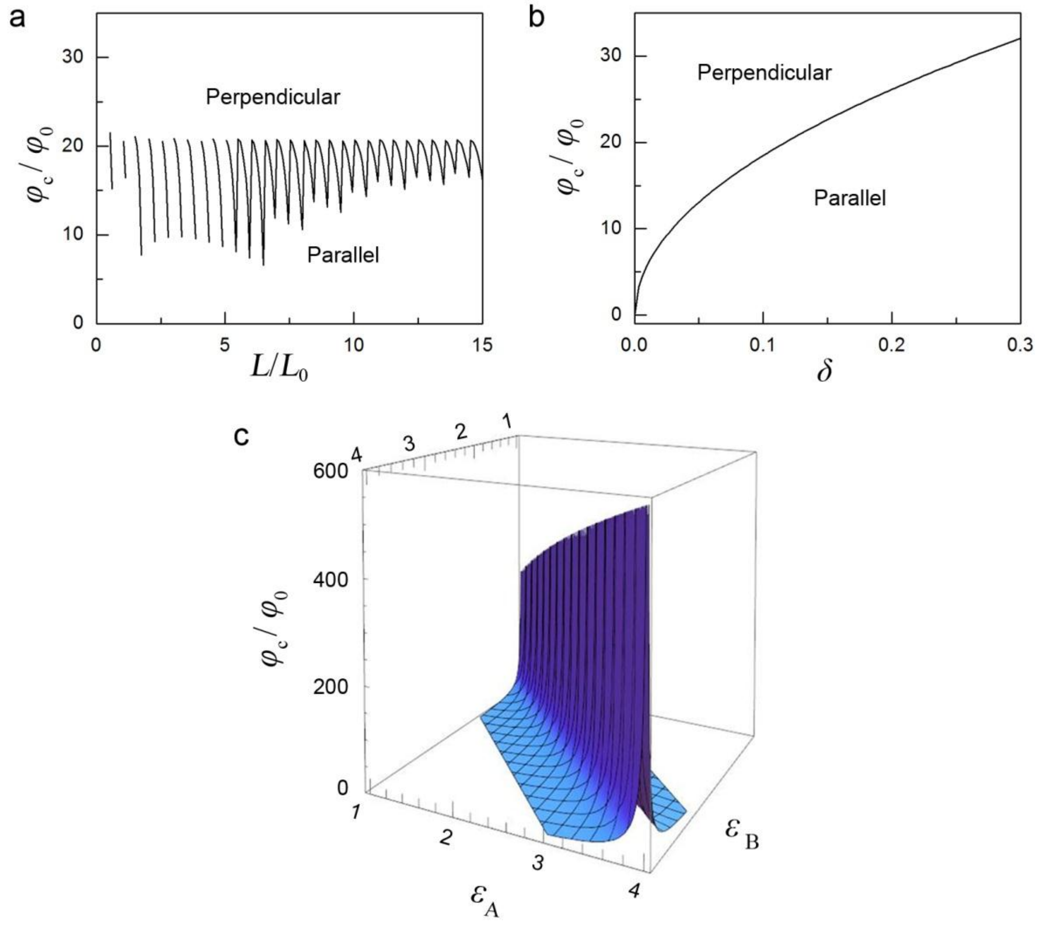

Supplementary Figure 18 | Relationship between the threshold of surface potential and its related parameters. Plots of φ_c/φ_0 vs. (a) L/L_0, (b) δ, (c) ε_A and ε_B, atop which perpendicular orientation is favoured and parallel orientation is favoured otherwise. Parameter values used in the diagrams are: ε_s = 3.9 (SiO_2), L_0 = 52.8 nm, and (a) ε_A = 2.6 (PS), ε_B = 3.0 (PMMA), δ = 0.133 (γ_{PS} = 29.9 mN m^{-1}, γ_{PMMA} = 30.02 mN m^{-1}, $\gamma_{PS\text{-}SiO_2}$= 177.41 mN m^{-1}, $\gamma_{PMMA\text{-}SiO_2}$ = 176.68 mN m^{-1}, $\gamma_{PS\text{-}PMMA}$ = 3.2 mN m^{-1}); (b) ε_A = 2.6 (PS), ε_B = 3.0 (PMMA), L/L_0 = 1; (c) δ = 0.133, L/L_0=1, $0.01 \le |\varepsilon_A - \varepsilon_B| \le 1$.

Supplementary Tables

Supplementary Table 1 | Summary of electric field strength that are used to align BCP.

No.	BCP type	Electric Field ($V\ \mu m^{-1}$)	Reference
1	PS-*b*-PMMA	3	Science, 1996, 273, 931-933
2	PS-*b*-PMMA	10	Macromolecular, 2000, 33, 3250-3253
3	PS-*b*-PMMA	40	Macromolecular, 2003, 36, 6178-6182
4	PS-*b*-PI et. al	5	Macromolecular, 2015, 36, 6178-6182
5	PS-*b*-PI	1	ACS Nano, 2013, 7, 3854-4867
6	PS-PHEMA-PMMA	15	Soft Matter, 2006, 2, 1089-1094;<br>ACS Nano, 2009, 3,1092-1096

Supplementary Table 2 | Contact angles of dewetted PS or PMMA homopolymer on e-beam irradiated substrates.

Substrate	PS (°)	PMMA (°)
SiO_2/Si	35.2 ± 0.5	~0
PI	17 ± 0.4	28.7 ± 0.5
NOS	32.3 ± 0.3	32.9 ± 0.5
SiO_2/Si prior to e-beam irradiation	11.6 ± 0.3	~0

To measure the contact angles, PS (molecular weight, 30k g mol^{-1}) and PMMA (molecular weight, 35k g mol^{-1}) homopolymers of ~20 nm were spin-coated on three different e-beam irradiated substrates and annealed at 200 ℃ for 30 h, forming dewetted PS or PMMA hemi-spherical droplets with height h and radius r. The contact angle θ is calculated by $\tan\frac{\theta}{2} = \frac{h}{r}$, where h and r were measured by AFM from at least 5 droplets. The irradiated substrates were prepared by Mode **3**. For an irradiation size of $2.4 \times 1.8\ mm^2$, the irradiation time for SiO_2/Si, PI, and NOS substrate were 40, 20, and 40 min, respectively. As the PMMA homopolymer shows strong affinity to the SiO_2/Si substrate, the PMMA wets the pristine SiO_2/Si and charged SiO_2/Si substrates and cannot form hemispherical droplets on them. Thus, the contact angle of PMMA homopolymer on both pristine SiO_2/Si and charged SiO_2/Si substrates were not exactly obtained and are considered as 0 (with a slight difference).

Supplementary Discussion

Dielectric constant

Dielectric constant of diblock copolymer is theoretically estimated from the permittivity of the monomer polymer of the two blocks. For diblock copolymer A–B, in which total molecular weight ratio of each monomer is f and $1 - f$, the dielectric constant of the block copolymer can be roughly estimated by

$$\varepsilon_{AB} = f\varepsilon_{A} + (1 - f)\varepsilon_{B} \tag{1}$$

where ε_{AB}, ε_{A}, and ε_{B} are relative dielectric constants of copolymer A–B, monomer A, and monomer B, respectively. For PS-*b*-PMMA, ε_{PS} = 2.6 (1 kHz) and ε_{PMMA} = 3.0 (1 kHz)[1]. Thus, $\varepsilon_{PS\text{-}b\text{-}PMMA}$ = 2.72 (46k-*b*-21k) or 2.80 (53k-*b*-54k).

Electric field strength

The electric field E generated from the electret can be derived according to Gauss's law and Kirchhoff's second law[2]. To be simplified, here we discuss only diblock copolymer A–B self-assembled on the charged planar electret substrate, whose area is S and lateral dimension ($\sim\sqrt{S}$) is much larger than its thickness D and the thickness of polymer film L. Under this condition, one has

$$\varphi = \frac{\sigma D}{\varepsilon_0 \varepsilon_s} \tag{2}$$

$$E = \frac{\varphi}{L + D\varepsilon_{AB}/\varepsilon_s} \tag{3}$$

where φ is the surface potential measured at the electret, σ is the planar density of charge, ε_0 is the permittivity of vacuum, ε_s is the relative dielectric constant of substrate.

In the instance presented in Figure 3, where PS-*b*-PMMA microdomain orientates on the charged SiO_2/Si electret, φ = 300 mV, D = 2.3 nm, $\varepsilon_s = \varepsilon_{SiO_2} = 3.9$, and $\varepsilon_{AB} = 2.8$. Therefore, for the PS-*b*-PMMA film with L = 30–200 nm, the generated electric field strength is 1.5–9.5 V μm^{-1} through the film. The electric field is comparable to the external electric fields that have been applied to align the BCP (see Supplementary Table 1).

We additionally note that because a planar electret model is applied, Supplementary Equations 2 and 3 may not be exactly valid for superfine nanopatterns shown in Figure 5. Estimation of E in these conditions would be much more complicated. Nevertheless, because the charge density in the patterns was similar, E upon the irradiated area should be close to that of the planar electrets.

Wetting property

To study the wetting property of PS-*b*-PMMA film on e-beam irradiated substrates, we compare the interfacial energies $\gamma_{s\text{-}p}$ between the PS or PMMA homopolymer and the irradiated substrates. In general, $\Delta\gamma = |\gamma_{s\text{-}PS} - \gamma_{s\text{-}PMMA}|$ can be utilized to indicate the wetting property of PS-*b*-PMMA film on a certain substrate[3]. For $\Delta\gamma \approx 0$, the substrate is considered non-preferential, i.e., neutral to both blocks, and can lead to a perpendicular orientation of PS-*b*-PMMA film; otherwise, the substrate is considered preferential to PS or PMMA block and would lead to a parallel orientation of PS-*b*-PMMA. According to the Young's equation $\gamma_{s\text{-}p} = \gamma_s - \gamma_p\cos\theta_p$, where γ_s and γ_p is the surface tension of the substrate and homopolymer and θ_p is the contact angle of the homopolymer on the substrate, one has $\Delta\gamma = |\gamma_{PS}\cos\theta_{PS} - \gamma_{PMMA}\cos\theta_{PMMA}|$. This formula can be simplified to $\Delta\gamma = \gamma_{PS}|\cos\theta_{PS} - \cos\theta_{PMMA}|$ as $\gamma_{PS} \approx \gamma_{PMMA}$[3]. Therefore, we can analyse the surface wetting property simply by comparing $\cos\theta_{PS}$ and $\cos\theta_{PMMA}$.

We have measured θ_{PS} and θ_{PMMA} on three electrets after e-beam irradiation and summarized the data in Supplementary Table 2. It is seen that $\Delta\gamma$ is very large on SiO_2/Si (θ_{PS} = 35.2°, $\theta_{PMMA} \approx 0$), relatively large on PI (θ_{PS} = 17.0°, θ_{PMMA} = 28.7°), and approximately zero on NOS (θ_{PS} = 32.3°, θ_{PMMA} = 32.9°). Therefore, after e-beam irradiation, SiO_2/Si is PMMA-preferential, PI is PS-preferential, and NOS is non-preferential to PS and PMMA blocks. Irrespective of the preferential wetting properties of the irradiated substrates, we can achieve perpendicular orientation of PS-*b*-PMMA film on all these substrates, demonstrating that interfacial energy is not or at least not the main driving force to control the microdomain orientation of PS-*b*-PMMA film.

Threshold of surface potential

Critical surface potential φ_c is the threshold of surface potential φ at the surface of the electret that generates E strong enough to align BCP perpendicularly orientated on the substrate. E is closely related to surface potential φ following Supplementary Equation 3. One obtains φ_c by solving

$$\Delta F_e + \Delta F_p = 0 \tag{4}$$

where ΔF_e and ΔF_p is the electrostatic energy difference and polymer free energy difference (elastic free energy of the stretched polymer chains and various interfacial energies) between parallel and perpendicular orientations. For a lamella diblock copolymer A–B (in a strong-segregation regime) self-assemblied on an electret of area S, where $\sqrt{S} >> L + D$, and A and B wet the substrate and air (in PS-*b*-PMMA, A = PMMA and B = PS), respectively, ΔF_e and ΔF_p are approximated by

$$\Delta F_e = -\frac{(\varepsilon_A - \varepsilon_B)^2}{4(\varepsilon_A + \varepsilon_B)}\varepsilon_0 \int_V E^2 \mathrm{d}V \tag{5}$$

$$\Delta F_{\mathrm{p}} = \frac{1}{3} S F_0 \left(\frac{\delta}{L/L_0} + 3 - \lambda^2 - \frac{2}{\lambda}\right) \tag{6}$$

where E is given by Supplementary Equation 3, L_0 is the lamella domain space at the equilibrium state, $\delta = \frac{\gamma_{\mathrm{A}} - \gamma_{\mathrm{B}} + \gamma_{\mathrm{s\text{-}B}} - \gamma_{\mathrm{s\text{-}A}}}{2\gamma_{\mathrm{A\text{-}B}}}$ is the mismatch of interfacial energies, $\lambda = \frac{2L/L_0}{[2L/L_0]}$ (the function $[x]$ donates the greatest integer less than or equal to x), and F_0 is the minimum free energy of the diblock polymer (including interfacial energy and entropic penalty but excluding contribution from electric field).[4]

Combining Supplementary Equations 3–6 and taking $\varepsilon_{\mathrm{AB}} = (\varepsilon_{\mathrm{A}} + \varepsilon_{\mathrm{B}})/2$ in the lamellar diblock copolymer, one obtains

$$\varphi_{\mathrm{c}} = \varphi_0 \frac{\sqrt{\varepsilon_{\mathrm{A}} + \varepsilon_{\mathrm{B}}}}{|\varepsilon_{\mathrm{A}} - \varepsilon_{\mathrm{B}}|} \sqrt{\frac{D}{L}\left(\frac{\delta}{L/L_0} + 3 - \lambda^2 - \frac{2}{\lambda}\right)\left(\frac{2L}{D} + \frac{\varepsilon_{\mathrm{A}} + \varepsilon_{\mathrm{B}}}{\varepsilon_{\mathrm{s}}}\right)} \tag{7}$$

where $\varphi_0 = \sqrt{\frac{F_0 D}{3\varepsilon_0}}$.

To understand the effect of the factors (L, δ, ε_{A}, and ε_{B}) on the electret-induced orientation method, we estimated the dependence of potential threshold on these parameters. Using suitable parameter data of BCP film self-assembled on SiO_2/Si substrate, we plot $\varphi_{\mathrm{c}}/\varphi_0$ as the function of L/L_0, δ, ε_{A}, and ε_{B}, respectively, in Supplementary Fig. 18.

It is evident from Supplementary Fig. 18 that the contrast of dielectric constants, i.e. $\Delta\varepsilon = |\varepsilon_{\mathrm{A}} - \varepsilon_{\mathrm{B}}|$, influences φ_{c} most significantly, especially at $\Delta\varepsilon \sim 0$. It is therefore expected that copolymers with larger $\Delta\varepsilon$ will be aligned by the electret-created field more easily. In fact, $\Delta\varepsilon$ of PS-*b*-PMMA is among the largest $\Delta\varepsilon$ of diblock copolymers ($\Delta\varepsilon_{\mathrm{PS\text{-}PMMA}} = 2.15$, $\Delta\varepsilon_{\mathrm{PS\text{-}P2VP}} = 1.6$, $\Delta\varepsilon_{\mathrm{PS\text{-}PI}} = 0.42$, $\Delta\varepsilon_{\mathrm{PS\text{-}PDMS}} = 0.17$ @ 0.1 Hz, 41 ℃)[5]. The influences from film thickness L and

interfacial energy mismatch δ, by contrast, are relatively small. It is predicted that the electret induced orientation would be possible over a wide film thickness, as long as the model discussed above is valid. The contribution from interfacial energy differences is also insignificant.

The above discussion is based on lamellar-forming morphology. Analysis for cylinder-forming morphology would be more complex; however, general dependence rules are similar. E-beam irradiation is feasible to yield surface potential on the electret larger than the threshold and generate electric field to overcome the interfacial energy barrier for perpendicular orientation.

Supplementary References

1 Brandrup, J., Immergut, E. H. & Grulke, E. A. *Polymer Handbook*, 4th edn. Wiley-Interscience (2003).

2 Sessler, G. M. *Electrets*, 2nd edn. Springer Berlin Heidelberg (1987).

3 Mansky, P., Liu, Y., Huang, E., Russell, T. P. & Hawker, C. J. Controlling polymer-surface interactions with random copolymer brushes. *Science* **275**, 1458-1460 (1997).

4 Ashok, B., Muthukumar, M. & Russell, T. P. Confined thin film diblock copolymer in the presence of an electric field. *J. Chem. Phys.* **115**, 1559-1564 (2001).

5 Kathrein, C. C., Kipnusu, W. K., Kremer, F. & Böker, A. Birefringence analysis of the effect of electric fields on the order–disorder transition temperature of lamellae forming block copolymers. *Macromolecules* **48**, 3354-3359 (2015).
